# A new approach for interpreting Random Forest models and its application to the biology of ageing

**DOI:** 10.1093/bioinformatics/bty087

**Published:** 2018-02-16

**Authors:** Fabio Fabris, Aoife Doherty, Daniel Palmer, João Pedro de Magalhães, Alex A Freitas

**Affiliations:** 1 School of Computing, University of Kent, Canterbury, Kent, UK; 2 Integrative Genomics of Ageing Group, Institute of Ageing and Chronic Disease, University of Liverpool, Liverpool, UK

## Abstract

**Motivation:**

This work uses the Random Forest (RF) classification algorithm to predict if a gene is over-expressed, under-expressed or has no change in expression with age in the brain. RFs have high predictive power, and RF models can be interpreted using a feature (variable) importance measure. However, current feature importance measures evaluate a feature as a whole (all feature values). We show that, for a popular type of biological data (Gene Ontology-based), usually only one value of a feature is particularly important for classification and the interpretation of the RF model. Hence, we propose a new algorithm for identifying the most important and most informative feature values in an RF model.

**Results:**

The new feature importance measure identified highly relevant Gene Ontology terms for the aforementioned gene classification task, producing a feature ranking that is much more informative to biologists than an alternative, state-of-the-art feature importance measure.

**Availability and implementation:**

The dataset and source codes used in this paper are available as ‘Supplementary Material’ and the description of the data can be found at: https://fabiofabris.github.io/bioinfo2018/web/.

**Supplementary information:**

Supplementary data are available at *Bioinformatics* online.

## 1 Introduction

In this work, we focus on predicting genes with altered expression with age in the brain. It has been commonly observed that there is an overall decline in neural function with age (Gustavsson *et al.*, 2011), and there is growing evidence that ageing plays a significant role in the development of degenerative diseases (Mattson and Magnus, 2006). The likelihood of developing neurodegenerative diseases such as Parkinson’s and Alzheimer’s dramatically increases with age (Mattson and Magnus, 2006). This is clearly important, as neurodegenerative diseases have a high social-economic impact, costing 146 billion Euros in 2004 in 28 surveyed European countries (Gustavsson *et al.*, 2011).

To study ageing processes in the brain holistically, we use a Random Forest (RF) classification algorithm (Breiman, 2001) to induce from data a model to predict if a given gene is over-expressed, under-expressed or have no change in expression with age in the brain. The RF algorithm is very popular in machine learning and bioinformatics (Touw *et al.*, 2013) due to its high predictive accuracy and the use of variable importance measures (VIMs). These measures allow us to identify the most important variables for classification in the model (a set of partly random decision trees) built by the RF algorithm.

However, current VIMs have an important limitation: they measure the importance of a variable as a whole, using all values taken by the variable. Sometimes, however, it is only one value of the variable (feature) that is important for classification, which requires a fine-grained measure of feature importance. This is the case in the dataset analysed in this work, which has 7490 features taking either a positive or negative value, representing the presence or absence (respectively) of a Gene Ontology (GO) term annotation for a gene (instance to be classified). As discussed in detail later, in general, the positive value of a GO term feature is much more informative and reliable than the negative value of that feature, since negative values represent lack of evidence and do not suggest any particular property for a gene. Hence, we propose a new method for measuring the importance of positive feature values, rather than the importance of a feature as a whole (both positive and negative values).

As related work, Hsing *et al.* (2008) use GO terms as features and a tree-based classification ensemble algorithm (boosting trees) to predict whether or not a protein is a hub in a network. In addition, Barardo *et al.* (2017) use GO terms as features and an RF algorithm to predict whether or not a chemical compound will increase *Caenorhabditis elegan’*s lifespan. These works rank features based on a measure of feature importance, but they ignore the difference between positive and negative feature values, which is precisely the limitation that we are addressing here.

It should be noted that GO terms are a very popular type of feature for classification in bioinformatics; and there are also several other types of binary features whose positive values tend to be much more important than negative values, like pathway annotations (e.g. KEGG pathway features), protein–protein interaction features, etc. (Fabris *et al.*, 2017). Hence, the proposed method for positive feature value evaluation has wider applicability in many other classification datasets in bioinformatics.

This paper’s main contribution is a new measure of feature value importance for RFs. This measure focuses only on positive feature values (ignoring negative values), and it is computed by a new algorithm that measures the predictive accuracy of a positive feature value by its overall predictive accuracy across all rules (root-to-leaf paths) in the RF where that feature value occurs. As a second contribution, we created a new dataset for studying gene expression with age in the brain and interpreted an RF model built from this dataset, based on the biology of ageing literature.

The remainder of this paper is organized as follows: Section 2 presents background on RFs and feature importance measures. Section 3 describes the creation of the dataset used in our experiments. Section 4 introduces the new measure of feature value importance. Section 5 reports the computational results and a biological interpretation of the most important GO terms based on the proposed measure of feature value importance. Finally, Section 6 presents the conclusions and some future work.

## 2 Background

### 2.1 Random Forest

The RF algorithm, which is widely used for classification in bioinformatics, builds *nTree* (a parameter) Random Trees (RT) during its training phase. This involves randomizing the training set in two ways for each RT: first, the training set is re-sampled with replacement, maintaining the original size of the dataset. The new re-sampled training set contains, on average, about 66% of unique instances (genes) from the original dataset. The set of training instances for a given RT is the ‘In-Bag’ instance set for that RT. The other 33% of the original dataset, which is not used for training, is the Out-Of-Bag (OOB) instance set for that RT.

As a second source of randomness for building an RT, the search for the best feature to split the set of instances at each RT node considers a randomly chosen feature subset of size *mtry* (a parameter), typically much smaller than the original feature set’s size. The instances at the current node are then split into two subsets according to a condition based on the values of the selected feature, creating two child nodes. This split aims to increase the similarity of classes within each instance subset and to decrease class similarity across the subsets. Next, the algorithm recurses in each instance subset until a stopping criterion is met.

In the prediction phase, a testing instance *t* is presented to each RT. For every RT, the feature values of *t* are matched against the feature-value conditions in the branches of the RT from the root node downwards, until *t* is assigned to a leaf node which predicts, for *t*, the most frequent class in that leaf node. The predictions of all RTs are combined (by voting) to output the RF’s final prediction.

RFs are difficult to interpret: they comprise many RTs making, to some extent, conflicting predictions; due to their randomized nature. However, feature importance measures can be used to find the most important features for classification in RF models, as discussed next.

### 2.2 Measures of feature importance in RFs

Several measures of feature (or variable) importance for RFs have been proposed, such as the Gini Variable Importance Measure (GVIM) (https://www.stat.berkeley.edu/%7Ebreiman/Using_random_forests_v4.0.pdf, accessed in 24/10/2017), Permutation VIM (PVIM) (Breiman, 2001), Conditional Permutation VIM (CPVIM) (Strobl *et al.*, 2008), Variable Selection using Random Forests (varSelRF) (Díaz-Uriarte and Alvarez de Andrés, 2006) and Variable Selection based on Minimal Distance (varSelMD) (Ishwaran *et al.*, 2010).

In essence, GVIM calculates each feature’s importance by averaging the OOB Gini impurity decrease when using the feature in a split of an RT node. PVIM calculates the average predictive accuracy difference, across all RTs, of the RF before and after permutating a given feature with a randomly selected one. CPVIM works similarly to PVIM, but considers conditional relationships among variables. VarSelRF iteratively removes features from the RF until its predictive accuracy is significantly reduced. Next, it returns the smallest set of features with predictive accuracy statistically equivalent to the best RF. Finally, varSelMD calculates the average depth of the features in the RF, assigning greater importance to features that are closer to the root node of an RT.

In a very recent work (Epifanio, 2017), the Intervention in Prediction Measure (IPM) was proposed and compared against the five above feature importance measures. That work concluded that IPM was superior to identify the most important features. Thus, we use the state-of-the-art IPM measure as a strong baseline measure in our experiments.

The IPM first computes, for each RT and each Out-Of-Bag (OOB) instance, a vector of size *J* (the number of features) containing in each *j*-th position the number of times the *j*-th feature was used to classify the instance. Next, this vector is normalized by dividing the frequency of use in each position by the summation of the frequencies over all *J* positions. This normalized vector (*V_n_*) contains the relative importance of each feature, i.e. its relative frequency of use to classify the instance. The vector *V_n_* is averaged across all OOB instances of interest and across all RTs to return the final IPM value for each feature.

Under the assumption that instances with different classes are classified using different features, these differences are reflected in the features’ IPM scores. Features that are important to classify instances of some class but are not so important to classify instances of other classes are of particular interest, since they are good predictors of a given class.

Note also that all the above importance measures evaluate a feature as a whole (i.e. all values of the feature), which is an important limitation in datasets where just one value of a feature has a good predictive power. Actually, in our dataset, one of the two values of each binary feature is much more interesting, as discussed in Section 4.2.

## 3 Dataset preparation

### 3.1 Collection of data about genes and classes

Age-related brain gene expression was collected from GEO and AgeMap. First, in AgeMap, all brain gene expression data was obtained by combining cerebellum, cerebrum, hippocampus and striatum expression datasets into one dataset (Zahn *et al.*, 2007). This gene expression data is already normalized with background subtracted. In total from this resource, gene expression data for 118 brain samples and 6712 mouse genes were extracted. Second, gene expression datasets and series datasets reporting expression levels in different ages or development stages in mammals’ brains were identified by searching GEO (Barrett *et al.*, 2006). Unsuitable datasets were removed. For example, custom datasets that examined a single pathway, specific diseases, mutants and treatments were excluded. Within the remaining 28 datasets, only age-related data from healthy, adult and non-treated samples were analysed. For example, in disease studies, we only took the controls at different age groups, and not the diseased state. Since ageing gene expression profiles can be detected early in adult life, all datasets with more than two adult time points were included, even if the oldest animals were middle-aged. In summary, 28 ageing-related GEO datasets and series comprising 1212 samples and a differing number of genes per dataset were obtained.

For both the GEO and AgeMap datasets, genes with more than 30% missing gene expression data across all samples were removed. Otherwise, null values were replaced by the probe’s average and probes targeting the same gene were averaged. Although we cannot perform a comprehensive evaluation of the quality of each experiment, our aggregation procedure is, in itself, a technique to cope with poor quality data. To identify genes that were consistently over- and under-expressed with age across all 31 datasets (3 from AgeMap and 28 from GEO), we found the genes with the largest number of putatively age-related signals in our multiple datasets, following the method described in (De Magalhães *et al.*, 2009). Human homologs for all mouse and rat protein-coding genes were downloaded from NCBI BioMart v87. High confidence one-to-one orthologs were extracted, and for each gene outputted from regression analysis, orthologs were identified. Finally, the proportion of human protein-coding genes within each class is 2.4%, 0.8% and 96.8% for the classes ‘over-expressed’, ‘under-expressed’ and ‘no change of expression’ with age in the brain, respectively.

### 3.2 GO term-based features

The instances (genes) are described by features representing the presence or absence of a GO term. We use GO term features because they are very well-known and easy to interpret––they use a controlled vocabulary, curated by experts, so the terms have well-defined biological meanings.

To retrieve the list of GO terms associated with our instances (genes), we have used the GO annotations from the XML file exported by the NCBI web page http://www.ncbi.nlm.nih.gov/gene (downloaded on the 18th of April 2017). This XML file was generated by the query:

‘ *Homo sapiens* ’[Organism] AND

(‘source_genomic’ [properties] AND

‘genetype protein coding’[Properties] AND

alive[prop])

The Gene Ontology definition (retrieved on the 14th of March 2017) was downloaded using the link http://geneontology.org/page/download-ontology#go-basic.obo.

Since a GO term implies all its ancestors (defining an ‘is-a’ hierarchy), we have expanded the set of GO terms annotating each instance (gene) to contain all ancestors of those GO terms. Also, we have eliminated GO terms annotating less than 10 instances, to avoid terms with little statistical support. This resulted in a dataset with 17 716 genes (instances) and 7490 GO terms (features). We also added to the dataset a numerical feature whose value is the total number of GO terms annotated for a gene.

## 4 Materials and methods

### 4.1 Experimental methodology

To measure predictive accuracy we use the popular Area Under the Receiver Operating Characteristic curve (AUROC), which is a plot of a classifier’s (here an RF model’s) True Positive Rate (TPR) as a function of its False Positive Rate (FPR). These rates are computed for each class by thresholding the class probabilities output by the RF using thresholds in the range [0, 1]. Each threshold produces a TPR and an FPR value, i.e. a point in the ROC curve. To obtain a single accuracy measure from the curve, we calculate the area under the ROC curve (AUROC) (Boyd *et al.*, 2013). The AUROC is calculated considering each class in turn as the ‘positive class’, and the final AUROC is the weighted average over the three classes, weighted by the number of instances in each class. AUROC values of 1.0, 0.5 and 0 indicate, respectively, a perfect classifier (all instances correctly predicted), a classifier with random guessing performance and the worst possible classifier (all instances wrongly predicted).

The AUROC is computed using the well-known 10-fold cross-validation procedure (Japkowicz and Shah, 2011). This method first divides the dataset into 10-folds of similar sizes. Next, each fold is temporarily removed from the dataset, one at a time, then the other 9-folds are used for training and the held-out fold used as a testing set for measuring predictive accuracy. The AUROC is the mean accuracy over the 10 testing sets. The AUROC value reported later is the mean over 30 runs of 10-fold cross-validation, to get more stable results, considering the randomized nature of RFs.

In each fold of the (external) cross-validation procedure we have used an internal 5-fold cross-validation procedure (on the training set only) to optimize the two most important parameters of the RF algorithm: *mtry* (the number of randomly sampled candidate features for selecting a split feature in an RT node) and *nTree* (the number of RTs in the RF). We have tested all pairwise combinations of the *mtry* values in the set $\left\{\sqrt{J}*0.5,\sqrt{J},\sqrt{J}*2\right\}$ where *J* is the number of features in the dataset, and *nTree* values in the set {100, 200, 300}; and used the pair with highest predictive accuracy on the internal cross-validation as the parameter-value pair for that external cross-validation fold.

Our dataset is highly unbalanced towards the class ‘no change in expression (N)’ with age, which has many more instances than the classes ‘over-expressed (O)’ and ‘under-expressed (U)’ with age. This leads to RFs models that are overly-conservative when predicting the minority classes ‘O’ and ‘U’. To attenuate this, we have performed an under-sampling of instances with classes ‘N’ and ‘O’ in the *in-bag* set (used to train the RF). That is, instances of classes ‘N’ and ‘O’ were randomly deleted until the three classes have the same number of instances––i.e. the number of instances in class ‘U’. We have performed experiments with and without this under-sampling, as reported later.

In order to measure the importance of features in the RF model, we have used the whole dataset to induce a final RF model using the pair of *mtry* and *ntree* values most frequently selected across the 10 external cross-validation folds; which was the pair: *nTree* = 300, *mtry* = 43 ($\sqrt{7490}*0.5$). This final model was induced using the above-described under-sampling, since these produced overall better results.

### 4.2 Measuring the importance of positive feature values

To measure the importance of each positive feature value in a RF model, we propose the ‘Computing the Predictive Accuracy of Random Tree Rules with Positive (±) Feature Values’ (COMPACT + FV) Algorithm, described below. The main motivation for this algorithm is, when calculating the importance of a feature *f*, to consider only the IF-THEN rules in the RF that contain ‘positive values’ of the feature *f*, as defined below. Note that every root-to-leaf path in an RF forms an IF-THEN rule, where the set of conditions along the path is the IF part and the class predicted by the leaf is the THEN part of the rule.

For the binary features used in this work we define a *positive feature value* as the value representing the presence (rather than absence) of the biological property linked with the feature. We use Gene Ontology (GO) terms as binary features, so a positive (negative) feature value indicates that an instance (gene) *is (is not) annotated* with a given GO term.

Considering only positive feature values has two major motivations: (1) Positive feature values tend to have a much higher level of confidence than negative ones. This is because a positive feature value indicates that ‘there is evidence’ for a given GO term; whilst a negative feature value indicates a ‘lack of evidence’ for that GO term––note that lack of evidence is different from evidence of absence. (2) Positive feature values are much more informative than negative ones. This is because a positive value tells us an instance (gene) has a certain biological property (GO term); whilst a negative value does not tell us any property possessed by a gene.

Recall that, for each RT in an RF model, each non-leaf node represents a test based on the value of a feature, leading to two child nodes––each of them associated with a condition that an instance must satisfy to reach that node. These two children correspond to the ‘positive’ and ‘negative’ values of the feature in the parent node.

COMPACT + FV, presented in Algorithm 1, iterates over every RT in the RF and for every feature, it extracts every IF-THEN rule (if any) containing the positive value of that feature and uses that rule’s statistics to measure the feature’s importance. The accuracy statistics of a rule in an RT are calculated using its Out-Of-Bag (OOB) instances, i.e. the instances that were not used to train that RT. Algorithm 1 extracts from the RF two statistics for each feature *f* and class *c*: (a) $Cov_{f+c}$, the OOB coverage, i.e. the total number of OOB instances covered by rules containing the positive value of feature *f* that predict class *c*; and (b) $Hits_{f+c}$, the OOB hits, i.e. the total number of OOB instances correctly classified by rules containing the positive value of feature *f* that predict class *c*. Note that our importance measure (and also the IPM importance measure) cannot be calculated by analysing only the structure of RF: they also depend on the OOB instances of each RT.

**Algorithm 1** The COMPACT+FV algorithm

1: **procedure** COMPACT + FV(Forest, Features)

2:  Initialize the counters $Hits_{f+c}$ and $Cov_{f+c}$ with the value zero for every feature *f* and class *c*.

3:  **for** each feature *f* in ‘Features’ **do**

4:   **for** each tree *t* in the Forest **do**

5:    Get all root-to-leaf rules in *t* with the positive value of *f*.

6:    **for** every such rule, *r***do**

7:     Get the class that *r* predicts (class *c*), the number of OOB instances that *r* covers (*cov*) and the number of correctly classified OOB instances (*hits*).

8:      Update the values of the *Cov* and *Hits* counters:

9:      $Hits_{f+c}\leftarrow Hits_{f+c}+hits$

10:      $Cov_{f+c}\leftarrow Cov_{f+c}+cov$

11:    **end for**

12:   **end for**

13:   **for** every class *c***do**

14:    Compute the precision of the positive value of *f*:

15:    $Prec_{f+c}\leftarrow Hits_{f+c}/Cov_{f+c}$

16:   **end for**

17:  **end for**

18: **end procedure**

After Algorithm 1 finishes executing, all importance scores for every feature *f* and class *c* ($Prec_{f+c}$) in an RF are computed. Recall that $Prec_{f+c}$ is the *precision* of all rules containing the positive value of feature *f* that predict class *c*.

### 4.3 An example of the use of the COMPACT + FV algorithm

**Fig. 1.**
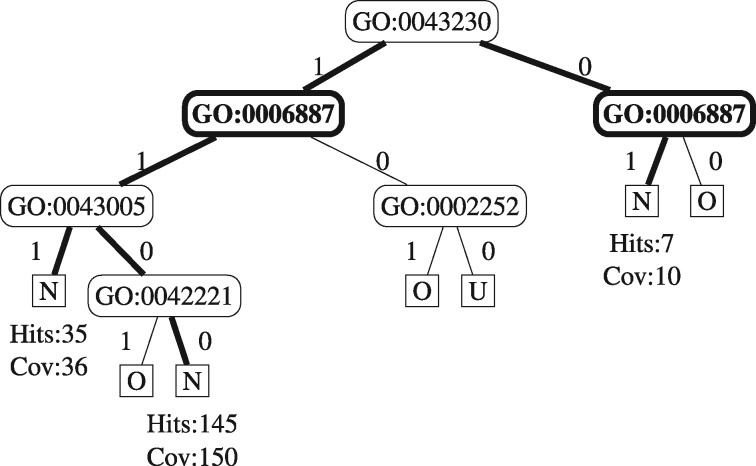
Example of a Random Tree used to calculate the statistics $Prec_{GO:0006887+N}$. In this tree, leaf nodes (where a prediction is made) are represented by squares with the predicted class in it, edges in bold form the relevant rules (a rule is a path from the root to a leaf node). We also show the OOB Hits and Coverages that are relevant to calculate the statistics $Prec_{GO:0006887+N}$

Next, we show the calculation of $Prec_{GO:0006887+N}$, i.e. the importance of a positive feature value representing the presence of the GO term ‘GO:0006887’ (exocytosis) to predict class ‘no change of expression’ with age in the brain (N). Let us assume that there are three rules in the RF that predict class ‘N’ using the positive value for feature ‘GO:0006887’. Next we present these rules using the following format: each rule contains conditions (RT nodes) involving a feature (where ‘1’ and ‘0’ denote the presence and absence of a GO term annotation, respectively) and in parenthesis the distribution of class frequencies of the OOB (Out Of Bag) instances that satisfied all conditions of the rule. After the OOB class distribution, we present the class predicted by the rule (the most frequent class in the in-bag instances used to build the RT). For pedagogical purposes, Figure 1 shows a fictitious RT that contains the same rules.

Rule 1: GO:0043230 = 1 AND GO:0006887 = 1 AND

  GO:0043005 = 1 (N: 35, U: 1): N

Rule 2: GO:0043230 = 1 AND GO:0006887 = 1 AND

  GO:0043005 = 0 AND GO:0042221 = 0

  (N: 145, O: 2, U: 3): N

Rule 3: GO:0043230 = 0 AND GO:0006887 = 1

  (N: 7, U: 3): N

Finally, to calculate the importance measure for feature ‘GO:0006887’ for class ‘N’ we must retrieve the rule-based coverages and hits: the first rule covers 36 OOB instances (genes), 35 of which were correctly classified (thus 35 hits). The second rule covers 150 OOB instances, 145 of which were correctly classified. The third rules cover 10 instances, 7 of which were correctly classified. So, the positive value of feature GO:0006887 has a rule-based precision of 0.9541 [the total rule-based hits divided by the total rule-based coverage: (35+145+7)/(36+150+10)].

## 5 Results and discussion

### 5.1 Predictive accuracy results

Table 1 shows the mean AUROC of the RF models per class and for all classes as a whole, with and without the in-bag under-sampling, across 30 runs of the 10-fold cross-validation procedure, as described earlier.

**Table 1.** Random Forest predictive accuracy results (AUROC) with and without under-sampling for the classes ‘Over-expressed (O)’, ‘Under-expressed (U)’ and with ‘No change in expression (N)’ with age in the brain, and the mean AUROC across classes (All) weighted by their number of instances

Training type	Classes			
	
	O	U	N	All
With under-sampling	0.758	0.676	0.707	0.708
Without under-sampling	0.733	0.653	0.698	0.699

As shown in Table 1, the RF using under-sampling has better predictive accuracy than the RF without under-sampling. In addition, inducing the RF model with under-sampling takes on average 3.8 h for each cross-validation run, which is much faster than the average 70.4 h to induce the RF model without under-sampling. This is due to the reduced in-bag set size when using under-sampling.

### 5.2 Feature importance results

Table 2 shows the most important GO terms based on the ranking by the proposed rule-based Precision measure. Table 3 shows the most important GO terms based on the ranking by the Intervention in Prediction measure (Section 2.2), a state-of-the-art measure of feature importance.

**Table 2.** Top-ranked GO terms (ranked by rule-based Precision) used to classify genes as ‘over-expressed’ and with ‘no change in expression’ with age in the brain

Rank	Feature i.d.	Feature name	Rule prec.	Rule hits
Top-ranked GO terms predicting class over-expressed with age				
1	GO:2001198	Regulation of dendritic cell differentiation	0.70	2.90
2	GO:0042605	Peptide antigen binding	0.49	5.80
3	GO:0042611	MHC protein complex	0.40	6.73
4	GO:0050431	Transforming growth factor beta binding	0.39	2.83
5	GO:0071294	Cellular response to zinc ion	0.36	7.97
6	GO:0071556	Integral component of lumenal side of endoplasmic reticulum membrane	0.36	6.45
7	GO:0071276	Cellular response to cadmium ion	0.33	5.07
8	GO:0002479	Antigen proc. and pres. of exogenous peptide antigen via MHC class I, TAP-dependent	0.32	14.57
9	GO:0042590	Antigen processing and presentation of exogenous peptide antigen via MHC class I	0.30	23.97
10	GO:0055038	Recycling endosome membrane	0.29	3.93
11	GO:0046686	Response to cadmium ion	0.28	4.73
12	GO:0060333	Interferon-gamma-mediated signaling pathway	0.27	35.73
13	GO:0044548	S100 protein binding	0.27	0.95
14	GO:0071402	Cellular response to lipoprotein particle stimulus	0.27	0.93
15	GO:0030670	Phagocytic vesicle membrane	0.26	5.07
16	GO:0019865	Immunoglobulin binding	0.26	1.13
17	GO:0012507	ER to Golgi transport vesicle membrane	0.23	10.27
18	GO:0030176	Integral component of endoplasmic reticulum membrane	0.23	5.20
Top-ranked GO terms predicting class no change in expression with age				
1	GO:0004930	G-protein coupled receptor activity	1.00	4480.70
2	GO:0006396	RNA processing	1.00	2688.77
3	GO:0050906	Detection of stimulus involved in sensory perception	1.00	2388.67
4	GO:0051606	Detection of stimulus	1.00	2287.60
5	GO:0050907	Detection of chemical stimulus involved in sensory perception	1.00	2237.87
6	GO:0009593	Detection of chemical stimulus	1.00	2079.60
7	GO:0004984	Olfactory receptor activity	1.00	1768.10
8	GO:0050911	Detection of chemical stimulus involved in sensory perception of smell	1.00	1624.60
9	GO:0005882	Intermediate filament	1.00	334.77
10	GO:0034470	ncRNA processing	1.00	302.03
11	GO:0006397	mRNA processing	1.00	301.43
12	GO:0031424	Keratinization	1.00	286.03
13	GO:0000151	Ubiquitin ligase complex	1.00	130.80
14	GO:0007608	Sensory perception of smell	1.00	112.77
15	GO:0032259	Methylation	1.00	110.87
16	GO:0016072	rRNA metabolic process	1.00	108.83
17	GO:0045095	Keratin filament	1.00	107.07
18	GO:0000375	RNA splicing, via transesterification reactions	1.00	99.90

*Note*: The columns contain: (1) the feature rank, (2) the feature identifier, (3) the feature name, (4) the mean rule-based Precision and (5) the mean rule-based Hits. Rule-based scores are based on the RF’s predictions on the Out-of-Bag datasets––not used for building the models. See the main text for definitions of Precision and Hits.

**Table 3.** Top-ranked GO terms [ranked by the Intervention in Prediction score (Epifanio, 2017)] used to classify genes as ‘over-expressed’, ‘under-expressed’ and with ‘no change in expression’ with age in the brain

Rank	Feature i.d.	Feature name	Interv. score
Top-Ranked GO terms predicting class over-expressed with age			
1	total	Number of GO annotations	1.22e–02
2	GO:0043005	Neuron projection	5.61e–03
3	GO:0097458	Neuron part	5.55e–03
4	GO:1903561	Extracellular vesicle	5.36e–03
5	GO:0070062	Extracellular exosome	5.33e–03
6	GO:0043230	Extracellular organelle	5.01e–03
7	GO:0044456	Synapse part	4.70e–03
8	GO:0002376	Immune system process	4.43e–03
9	GO:0042995	Cell projection	4.25e–03
10	GO:0044421	Extracellular region part	4.21e–03
11	GO:0031982	Vesicle	3.77e–03
12	GO:0044444	Cytoplasmic part	3.58e–03
13	GO:0002252	Immune effector process	3.45e–03
14	GO:0050896	Response to stimulus	3.07e–03
15	GO:0002682	Regulation of immune system process	2.72e–03
16	GO:0048731	System development	2.56e–03
Top-ranked GO terms predicting class under-expressed with age			
1	total	Number of GO annotations	1.30e–02
2	GO:0043005	Neuron projection	6.81e–03
3	GO:0097458	Neuron part	6.51e–03
4	GO:0044456	Synapse part	5.73e–03
5	GO:1903561	Extracellular vesicle	5.15e–03
6	GO:0070062	Extracellular exosome	5.11e–03
7	GO:0042995	Cell projection	4.82e–03
8	GO:0043230	Extracellular organelle	4.80e–03
9	GO:0044421	Extracellular region part	4.27e–03
10	GO:0002376	Immune system process	4.10e–03
11	GO:0031982	Vesicle	3.79e–03
12	GO:0044444	Cytoplasmic part	3.77e–03
13	GO:0050896	Response to stimulus	3.04e–03
14	GO:0048731	System development	2.92e–03
15	GO:0002252	Immune effector process	2.88e–03
16	GO:0007399	Nervous system development	2.84e–03
Top-ranked GO terms predicting class no change in expression with age			
1	total	Number of GO annotations	1.43e–02
2	GO:0097458	Neuron part	5.36e–03
3	GO:0043005	Neuron projection	5.31e–03
4	GO:1903561	Extracellular vesicle	4.85e–03
5	GO:0070062	Extracellular exosome	4.77e–03
6	GO:0043230	Extracellular organelle	4.55e–03
7	GO:0044456	Synapse part	4.43e–03
8	GO:0044421	Extracellular region part	4.05e–03
9	GO:0042995	Cell projection	4.03e–03
10	GO:0044444	Cytoplasmic part	3.97e–03
11	GO:0002376	Immune system process	3.91e–03
12	GO:0031982	Vesicle	3.66e–03
13	GO:0050896	Response to stimulus	3.21e–03
14	GO:0005515	Protein binding	2.98e–03
15	GO:0048731	System development	2.79e–03
16	GO:0008150	Biological_process	2.75e–03

*Note*: The columns are: (1) the feature’s rank, (2) the feature’s identifier, (3) the feature’s name and (4) the Intervention score. The ‘Total’ feature is the number of GO terms annotated for each gene.

Contrasting the two tables, it is clear that the rule-based Precision and the Intervention score lead to very different sets of top-ranked GO terms. Unfortunately, the intervention-based ranking is not useful for identifying GO terms that are strong predictors of a single class, since the top-ranked GO terms based on that score are very similar for all three classes.

This is despite the fact that this score was computed for instances of each class separately. This result is due to the fact that the Intervention score reflects the use of both positive and negative feature values. Actually, for most features in our dataset, the large majority of instances have a negative feature value. Hence, the negative value of a feature tends to contribute more to its Intervention score than its positive value. Since negative feature values are much less informative than positive ones (as discussed earlier), this has the undesirable effect of preventing the identification of positive feature values which are relatively rare but provide much more informative predictions for a given class.

In contrast, the rule-based Precision focuses on rules containing only positive feature values, without being distracted by negative values. As a result, this measure successfully identifies different sets of top-ranked GO terms for predicting different classes. In addition, in general, the GO terms in Table 2 (Precision-based ranking) describe more specific and more informative gene properties than the more generic (often very broad) GO terms in Table 3 (intervention-based ranking).

These results reflect the different biases of the two measures. The Intervention measure rewards mainly the high frequency of use of a feature in an RF, without explicitly rewarding predictive accuracy. This measure implicitly rewards accuracy, since highly accurate features tend to be used to classify more instances. However, since the negative value of a feature is used to classify many more instances than its positive value, the measure is biased towards rewarding features with accurate negative values, rather than accurate positive values. In contrast, the rule-based Precision measure rewards mainly the predictive accuracy of a positive feature value in an RF’s rules. The trade-off is that positive feature values have a relatively small frequency of use (see the Rule Hits column in Table 2); but this is overall a good trade-off, since the negative feature values are not very informative, as discussed earlier.

Hence, in the remainder of this section, we focus on the top-ranked GO terms identified by the rule-based Precision measure (Table 2). This table contains 18 top-ranked GO terms predicting the ‘over-expressed’ (O) and ‘no change with age’ (N) classes. There are 26 GO terms whose positive value has the maximum rule-based Precision of 1.0 when predicting the class ‘N’, we only show the top-18 in the table (sorted by the second criterion, the rule-based coverage). Most of these GO term annotations also have large numbers of rule-based Hits in the Out-of-Bag instances, as shown in the table, since this class has a prior probability (relative frequency) of 96.8%. The top-18 GO terms predicting class ‘O’ in the table have overall much lower rule-based Precision and Hits in the Out-of-Bag instances since this class has much fewer instances. However, these GO terms still have a rule-based Precision substantially higher than the prior probability of the class ‘O’, which is just 2.4%. The top-ranked GO terms predicting the ‘under-expressed’ class are not shown in this table because they have low rule-based Precision and Hits (this class’ prior probability is just 0.8%), so they are not reliable enough for further analysis.

As shown in Table 2, positive feature values of GO terms used to predict over-expression included immune response pathways, responses to heavy metal toxicity and endoplasmic reticulum membrane genes.

Over-expression of the immune response (including GO:2001198, rank 1; GO:0042605, rank 2 and GO:0042611, rank 3) is a commonly seen signature of the ageing transcriptome. Meta-analysis of ageing expression studies shows over-expression of immune response genes to be a consistent signature of ageing (De Magalhães *et al.*, 2009). This includes the over-expression of inflammation genes, representative of an ‘inflamm-ageing’ phenotype associated with numerous ageing related diseases such as Alzheimer’s disease and cancer (Xia *et al.*, 2016). S100 proteins (GO:0044548, rank 13) are also linked to inflammation response, with constitutive expression in neutrophils and interleukin-induced expression in other cells. These proteins have been associated with inflammation-related diseases and cancer, and possibly have a function in extracellular oxidant scavenging (Goyette and Geczy, 2011). Oxidative damage in the brain increases with age, including lipid peroxidation and protein carbonylation (Head *et al.*, 2002).

GO terms related to cadmium (GO:0071276, rank 7) and zinc ion (GO:0071294, rank 5) response predicting over-expression may be linked, since the toxicity of both metals is oxidative stress based, the former by depletion of thiol-based antioxidants (Cuypers *et al.*, 2010), while the latter causes copper deficiency, reducing the cells’ ability to produce copper based antioxidants such as superoxide dismutase (Paynter *et al.*, 1979).

Oxidised proteins may act as an intermediate to protein aggregate clusters, causing a breakdown of normal cellular function (Squier, 2001). The unfolded protein response (UPR), mediated by the endoplasmic reticulum (ER), produces chaperones and upregulates the inflammation response to deal with protein aggregation and misfolding (Cao and Kaufman, 2012). This response is driven by transmembrane proteins in the ER and Golgi apparatus, facilitating communication between these organelles and the nucleus, potentially explaining the use of related terms (GO:0071556, rank 6; GO:0012507, rank 17 and GO:0030176, rank 18) to predict over-expression.

Positive feature values of GO terms used to predict unchanged expression included receptor activity (including olfaction), RNA processing and structural genes, however, this is also the largest class and so there were many other categories with high precision. These categories are all very large, including genes involved in a wide variety of functions.

G-protein coupled receptor activity (GO:0004930, rank 1) is closely related to olfaction. Olfactory receptors are a subset of G-protein coupled receptor and several olfaction-related terms co-occur with GO:0004930, for instance ‘sensory perception of smell’ (GO:0007608, rank 14) ‘olfactory receptor activity’ (GO:0004984, rank 7) and ‘detection of chemical stimulus involved in the sensory perception of smell’ (GO:0050911, rank 8) (Binns *et al.*, 2009). Olfactory neurogenesis is reduced in aged mice, as is the ability to distinguish different odours, however, olfactory interneurons are increased (Enwere *et al.*, 2004). Further, ageing-related diseases such as AD are frequently associated with declined olfactory function (Attems *et al.*, 2005). In humans, the olfactory bulb appears to be the main benefactor of neuronal progenitor cells migrating from the lateral ventricle, suggesting it is more capable of neuroregeneration than other areas of the brain (Armstrong and Barker, 2001). Olfactory genes do not just relate to the sense of smell, but also to numerous other chemoreceptor mediated functions. For instance, OR51E2 mediates cytoskeletal remodelling and proliferation in airway smooth muscle cells, in response to short-chain fatty acids (Aisenberg *et al.*, 2016), while OR10J5 mediates angiogenesis and stimulates cellular migration (Kim *et al.*, 2015).

RNA processing and its child terms (including GO:0006396, rank 2; GO:0034470, rank 10 and GO:0006397, rank 11) is a huge category containing over 4000 annotations in humans. While there is no evidence that the category changes in expression with age, there is a sex difference in humans with the ageing male brain underexpressing RNA processing GO groups relative to females (Berchtold *et al.*, 2008). Likewise, the various structural GO groups highlighted are large and integral to basic cellular function. Intermediate filaments (GO:0005882, rank 9) play an important structural role in the brain, supporting axons and allowing an increase in axonal diameter (Fuchs and Cleveland, 1998). In addition, intermediate filaments including keratin filaments (GO:0045095, rank 17) have been implicated in numerous diseases, including cancer, and have possible roles in stress resistance and ageing (Hyder *et al.*, 2011).

## 6 Conclusion and future work

Existing measures of feature importance for RFs do not differentiate between positive (the presence of a property) and negative feature values (the lack of evidence for a property). This is an important limitation, as for many feature types used in bioinformatics, like the very popular Gene Ontology (GO) terms-based features used in this work, positive feature values are much more informative than negative values. This is because the presence of a property (like a GO term annotation) gives much more useful information about a gene than the absence of a property. In addition, negative feature values are less reliable because they encode absence of evidence, rather than evidence for the property’s absence.

For this reason, we have proposed a new feature importance measure that evaluates the precision (predictive accuracy) of only the positive feature values in an RF, without being unduly influenced by the negative feature values. This measure works by finding rules (root-to-leaf paths) in the RF that use the positive feature value to predict a class of interest and then measuring the combined precision of these rules.

We have compared the results of using our feature importance measure against a state-of-the-art feature importance measure (the Intervention in Prediction measure), on a dataset created to predict whether or not a gene is ‘over-expressed’, ‘under-expressed’ or has ‘no change in expression’ with age in the human brain, using Gene Ontology (GO) terms as features. We have contrasted the top-ranked GO terms based on the rankings produced by our rule-based Precision measure and the Intervention in Prediction measure, and have concluded that the most important GO terms based on the Precision measure are more useful (more informative) to study our ageing-related problem. As evidence for this, we presented an interpretation of the biological meaning of the top-ranked GO terms, according to the proposed rule-based Precision measure.

As future work, we plan to apply our feature importance measure to other human tissues, and use other feature types besides GO terms.

## Funding

This work was supported by a Leverhulme Trust research Grant (Ref. No. RPG-2016-015) to J.P.de.M. and A.A.F. 

*Conflict of Interest*: none declared.

## Supplementary Material

Supplementary Data
