# Supplementary figures and images for: A new approach for interpreting Random Forest models and its application to the biology of ageing (part 2 of 2)
Source: Bioinformatics. 2018 Feb 16;34(14):2449–56. doi: 10.1093/bioinformatics/bty087 (PMC6041990; doi:10.1093/bioinformatics/bty087)

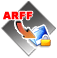

Supplement: Supplementary Data [file bty087_src_code_and_dataset.zip › src_code_and_dataset/java_src/weka/bin/weka/gui/beans/icons/DefaultDataSink_animated.gif]

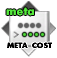

Supplement: Supplementary Data [file bty087_src_code_and_dataset.zip › src_code_and_dataset/java_src/weka/bin/weka/gui/beans/icons/MetaCost_animated.gif]

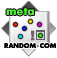

Supplement: Supplementary Data [file bty087_src_code_and_dataset.zip › src_code_and_dataset/java_src/weka/bin/weka/gui/beans/icons/RandomCommittee_animated.gif]

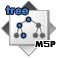

Supplement: Supplementary Data [file bty087_src_code_and_dataset.zip › src_code_and_dataset/java_src/weka/bin/weka/gui/beans/icons/M5P_animated.gif]

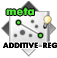

Supplement: Supplementary Data [file bty087_src_code_and_dataset.zip › src_code_and_dataset/java_src/weka/bin/weka/gui/beans/icons/AdditiveRegression.gif]

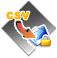

Supplement: Supplementary Data [file bty087_src_code_and_dataset.zip › src_code_and_dataset/java_src/weka/bin/weka/gui/beans/icons/CSVSaver.gif]

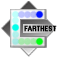

Supplement: Supplementary Data [file bty087_src_code_and_dataset.zip › src_code_and_dataset/java_src/weka/bin/weka/gui/beans/icons/FarthestFirst.gif]

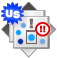

Supplement: Supplementary Data [file bty087_src_code_and_dataset.zip › src_code_and_dataset/java_src/weka/bin/weka/gui/beans/icons/filters.unsupervised.attribute.MakeIndicator.gif]

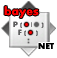

Supplement: Supplementary Data [file bty087_src_code_and_dataset.zip › src_code_and_dataset/java_src/weka/bin/weka/gui/beans/icons/BayesNet_animated.gif]

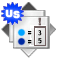

Supplement: Supplementary Data [file bty087_src_code_and_dataset.zip › src_code_and_dataset/java_src/weka/bin/weka/gui/beans/icons/filters.unsupervised.attribute.StringToWordVector_animated.gif]

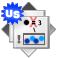

Supplement: Supplementary Data [file bty087_src_code_and_dataset.zip › src_code_and_dataset/java_src/weka/bin/weka/gui/beans/icons/filters.unsupervised.instance.RemoveWithValues.gif]

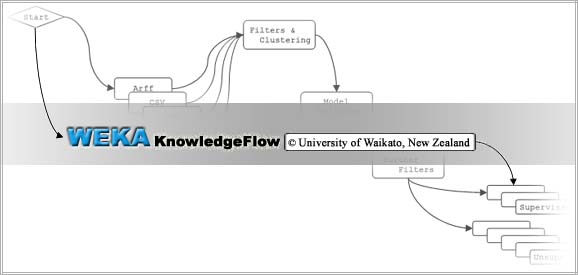

Supplement: Supplementary Data [file bty087_src_code_and_dataset.zip › src_code_and_dataset/java_src/weka/bin/weka/gui/beans/icons/splash.jpg]

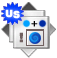

Supplement: Supplementary Data [file bty087_src_code_and_dataset.zip › src_code_and_dataset/java_src/weka/bin/weka/gui/beans/icons/filters.unsupervised.attribute.MergeTwoValues.gif]

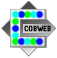

Supplement: Supplementary Data [file bty087_src_code_and_dataset.zip › src_code_and_dataset/java_src/weka/bin/weka/gui/beans/icons/Cobweb.gif]
