# Supplementary figures and images for: A new approach for interpreting Random Forest models and its application to the biology of ageing (part 1 of 2)
Source: Bioinformatics. 2018 Feb 16;34(14):2449–56. doi: 10.1093/bioinformatics/bty087 (PMC6041990; doi:10.1093/bioinformatics/bty087)

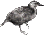

Supplement: Supplementary Data [file bty087_src_code_and_dataset.zip › src_code_and_dataset/java_src/weka/bin/weka/gui/weka_animated.gif]

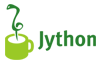

Supplement: Supplementary Data [file bty087_src_code_and_dataset.zip › src_code_and_dataset/java_src/weka/bin/weka/gui/scripting/images/jython_small.png]

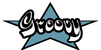

Supplement: Supplementary Data [file bty087_src_code_and_dataset.zip › src_code_and_dataset/java_src/weka/bin/weka/gui/scripting/images/groovy_small.png]

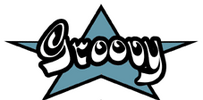

Supplement: Supplementary Data [file bty087_src_code_and_dataset.zip › src_code_and_dataset/java_src/weka/bin/weka/gui/scripting/images/groovy_medium.png]

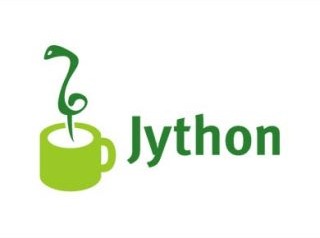

Supplement: Supplementary Data [file bty087_src_code_and_dataset.zip › src_code_and_dataset/java_src/weka/bin/weka/gui/scripting/images/jython_medium.png]

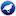

Supplement: Supplementary Data [file bty087_src_code_and_dataset.zip › src_code_and_dataset/java_src/weka/bin/weka/gui/weka_icon_new_small.png]

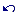

Supplement: Supplementary Data [file bty087_src_code_and_dataset.zip › src_code_and_dataset/java_src/weka/bin/weka/gui/images/undo.gif]

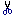

Supplement: Supplementary Data [file bty087_src_code_and_dataset.zip › src_code_and_dataset/java_src/weka/bin/weka/gui/images/cut.gif]

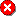

Supplement: Supplementary Data [file bty087_src_code_and_dataset.zip › src_code_and_dataset/java_src/weka/bin/weka/gui/images/error_small.gif]

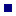

Supplement: Supplementary Data [file bty087_src_code_and_dataset.zip › src_code_and_dataset/java_src/weka/bin/weka/gui/images/stop.gif]

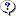

Supplement: Supplementary Data [file bty087_src_code_and_dataset.zip › src_code_and_dataset/java_src/weka/bin/weka/gui/images/question_small.gif]

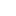

Supplement: Supplementary Data [file bty087_src_code_and_dataset.zip › src_code_and_dataset/java_src/weka/bin/weka/gui/images/empty.gif]

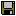

Supplement: Supplementary Data [file bty087_src_code_and_dataset.zip › src_code_and_dataset/java_src/weka/bin/weka/gui/images/save.gif]

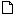

Supplement: Supplementary Data [file bty087_src_code_and_dataset.zip › src_code_and_dataset/java_src/weka/bin/weka/gui/images/new.gif]

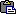

Supplement: Supplementary Data [file bty087_src_code_and_dataset.zip › src_code_and_dataset/java_src/weka/bin/weka/gui/images/paste.gif]

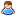

Supplement: Supplementary Data [file bty087_src_code_and_dataset.zip › src_code_and_dataset/java_src/weka/bin/weka/gui/images/user.png]

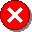

Supplement: Supplementary Data [file bty087_src_code_and_dataset.zip › src_code_and_dataset/java_src/weka/bin/weka/gui/images/error.gif]

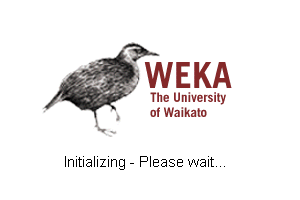

Supplement: Supplementary Data [file bty087_src_code_and_dataset.zip › src_code_and_dataset/java_src/weka/bin/weka/gui/images/weka_splash.gif]

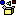

Supplement: Supplementary Data [file bty087_src_code_and_dataset.zip › src_code_and_dataset/java_src/weka/bin/weka/gui/images/objects.gif]

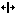

Supplement: Supplementary Data [file bty087_src_code_and_dataset.zip › src_code_and_dataset/java_src/weka/bin/weka/gui/images/resize.gif]

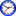

Supplement: Supplementary Data [file bty087_src_code_and_dataset.zip › src_code_and_dataset/java_src/weka/bin/weka/gui/images/history.png]

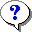

Supplement: Supplementary Data [file bty087_src_code_and_dataset.zip › src_code_and_dataset/java_src/weka/bin/weka/gui/images/question.gif]

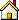

Supplement: Supplementary Data [file bty087_src_code_and_dataset.zip › src_code_and_dataset/java_src/weka/bin/weka/gui/images/home.gif]

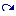

Supplement: Supplementary Data [file bty087_src_code_and_dataset.zip › src_code_and_dataset/java_src/weka/bin/weka/gui/images/redo.gif]

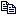

Supplement: Supplementary Data [file bty087_src_code_and_dataset.zip › src_code_and_dataset/java_src/weka/bin/weka/gui/images/copy.gif]

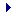

Supplement: Supplementary Data [file bty087_src_code_and_dataset.zip › src_code_and_dataset/java_src/weka/bin/weka/gui/images/run.gif]

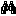

Supplement: Supplementary Data [file bty087_src_code_and_dataset.zip › src_code_and_dataset/java_src/weka/bin/weka/gui/images/find.gif]

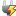

Supplement: Supplementary Data [file bty087_src_code_and_dataset.zip › src_code_and_dataset/java_src/weka/bin/weka/gui/images/connect.png]

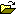

Supplement: Supplementary Data [file bty087_src_code_and_dataset.zip › src_code_and_dataset/java_src/weka/bin/weka/gui/images/open.gif]

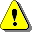

Supplement: Supplementary Data [file bty087_src_code_and_dataset.zip › src_code_and_dataset/java_src/weka/bin/weka/gui/images/information.gif]

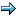

Supplement: Supplementary Data [file bty087_src_code_and_dataset.zip › src_code_and_dataset/java_src/weka/bin/weka/gui/images/forward.gif]

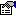

Supplement: Supplementary Data [file bty087_src_code_and_dataset.zip › src_code_and_dataset/java_src/weka/bin/weka/gui/images/properties.gif]

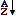

Supplement: Supplementary Data [file bty087_src_code_and_dataset.zip › src_code_and_dataset/java_src/weka/bin/weka/gui/images/sort.gif]

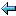

Supplement: Supplementary Data [file bty087_src_code_and_dataset.zip › src_code_and_dataset/java_src/weka/bin/weka/gui/images/back.gif]

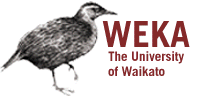

Supplement: Supplementary Data [file bty087_src_code_and_dataset.zip › src_code_and_dataset/java_src/weka/bin/weka/gui/images/weka_background.gif]

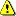

Supplement: Supplementary Data [file bty087_src_code_and_dataset.zip › src_code_and_dataset/java_src/weka/bin/weka/gui/images/information_small.gif]

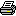

Supplement: Supplementary Data [file bty087_src_code_and_dataset.zip › src_code_and_dataset/java_src/weka/bin/weka/gui/images/print.gif]

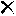

Supplement: Supplementary Data [file bty087_src_code_and_dataset.zip › src_code_and_dataset/java_src/weka/bin/weka/gui/images/delete.gif]

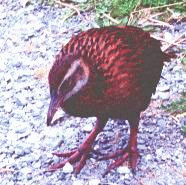

Supplement: Supplementary Data [file bty087_src_code_and_dataset.zip › src_code_and_dataset/java_src/weka/bin/weka/gui/weka3.gif]

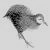

Supplement: Supplementary Data [file bty087_src_code_and_dataset.zip › src_code_and_dataset/java_src/weka/bin/weka/gui/weka_icon.jpg]

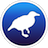

Supplement: Supplementary Data [file bty087_src_code_and_dataset.zip › src_code_and_dataset/java_src/weka/bin/weka/gui/weka_icon_new_48.png]

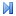

Supplement: Supplementary Data [file bty087_src_code_and_dataset.zip › src_code_and_dataset/java_src/weka/bin/weka/gui/beans/icons/resultset_last.png]

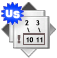

Supplement: Supplementary Data [file bty087_src_code_and_dataset.zip › src_code_and_dataset/java_src/weka/bin/weka/gui/beans/icons/filters.unsupervised.attribute.NumericToBinary_animated.gif]

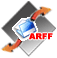

Supplement: Supplementary Data [file bty087_src_code_and_dataset.zip › src_code_and_dataset/java_src/weka/bin/weka/gui/beans/icons/DefaultDataSource_animated.gif]

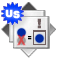

Supplement: Supplementary Data [file bty087_src_code_and_dataset.zip › src_code_and_dataset/java_src/weka/bin/weka/gui/beans/icons/filters.unsupervised.attribute.Remove_animated.gif]

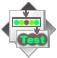

Supplement: Supplementary Data [file bty087_src_code_and_dataset.zip › src_code_and_dataset/java_src/weka/bin/weka/gui/beans/icons/TestSetMaker.gif]

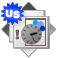

Supplement: Supplementary Data [file bty087_src_code_and_dataset.zip › src_code_and_dataset/java_src/weka/bin/weka/gui/beans/icons/filters.unsupervised.attribute.TimeSeriesDelta_animated.gif]

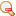

Supplement: Supplementary Data [file bty087_src_code_and_dataset.zip › src_code_and_dataset/java_src/weka/bin/weka/gui/beans/icons/zoom_out.png]

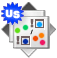

Supplement: Supplementary Data [file bty087_src_code_and_dataset.zip › src_code_and_dataset/java_src/weka/bin/weka/gui/beans/icons/filters.unsupervised.attribute.RandomProjection.gif]

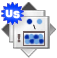

Supplement: Supplementary Data [file bty087_src_code_and_dataset.zip › src_code_and_dataset/java_src/weka/bin/weka/gui/beans/icons/filters.unsupervised.instance.SparseToNonSparse.gif]

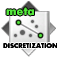

Supplement: Supplementary Data [file bty087_src_code_and_dataset.zip › src_code_and_dataset/java_src/weka/bin/weka/gui/beans/icons/RegressionByDiscretization.gif]

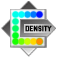

Supplement: Supplementary Data [file bty087_src_code_and_dataset.zip › src_code_and_dataset/java_src/weka/bin/weka/gui/beans/icons/MakeDensityBasedClusterer_animated.gif]

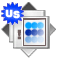

Supplement: Supplementary Data [file bty087_src_code_and_dataset.zip › src_code_and_dataset/java_src/weka/bin/weka/gui/beans/icons/filters.unsupervised.instance.RemoveFolds_animated.gif]

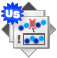

Supplement: Supplementary Data [file bty087_src_code_and_dataset.zip › src_code_and_dataset/java_src/weka/bin/weka/gui/beans/icons/filters.unsupervised.attribute.RemoveUseless_animated.gif]

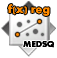

Supplement: Supplementary Data [file bty087_src_code_and_dataset.zip › src_code_and_dataset/java_src/weka/bin/weka/gui/beans/icons/LeastMedSq.gif]

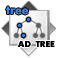

Supplement: Supplementary Data [file bty087_src_code_and_dataset.zip › src_code_and_dataset/java_src/weka/bin/weka/gui/beans/icons/ADTree_animated.gif]

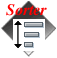

Supplement: Supplementary Data [file bty087_src_code_and_dataset.zip › src_code_and_dataset/java_src/weka/bin/weka/gui/beans/icons/Sorter_animated.gif]

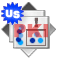

Supplement: Supplementary Data [file bty087_src_code_and_dataset.zip › src_code_and_dataset/java_src/weka/bin/weka/gui/beans/icons/filters.unsupervised.attribute.PKIDiscretize_animated.gif]

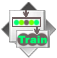

Supplement: Supplementary Data [file bty087_src_code_and_dataset.zip › src_code_and_dataset/java_src/weka/bin/weka/gui/beans/icons/TrainingSetMaker_animated.gif]

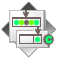

Supplement: Supplementary Data [file bty087_src_code_and_dataset.zip › src_code_and_dataset/java_src/weka/bin/weka/gui/beans/icons/ClassAssigner.gif]

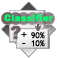

Supplement: Supplementary Data [file bty087_src_code_and_dataset.zip › src_code_and_dataset/java_src/weka/bin/weka/gui/beans/icons/ClassifierPerformanceEvaluator.gif]

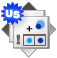

Supplement: Supplementary Data [file bty087_src_code_and_dataset.zip › src_code_and_dataset/java_src/weka/bin/weka/gui/beans/icons/filters.unsupervised.attribute.AddCluster_animated.gif]

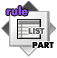

Supplement: Supplementary Data [file bty087_src_code_and_dataset.zip › src_code_and_dataset/java_src/weka/bin/weka/gui/beans/icons/PART_animated.gif]

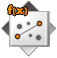

Supplement: Supplementary Data [file bty087_src_code_and_dataset.zip › src_code_and_dataset/java_src/weka/bin/weka/gui/beans/icons/Default_functionsClassifier.gif]

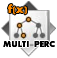

Supplement: Supplementary Data [file bty087_src_code_and_dataset.zip › src_code_and_dataset/java_src/weka/bin/weka/gui/beans/icons/MultilayerPerceptron.gif]

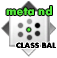

Supplement: Supplementary Data [file bty087_src_code_and_dataset.zip › src_code_and_dataset/java_src/weka/bin/weka/gui/beans/icons/ClassBalancedND.gif]

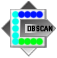

Supplement: Supplementary Data [file bty087_src_code_and_dataset.zip › src_code_and_dataset/java_src/weka/bin/weka/gui/beans/icons/DBScan.gif]

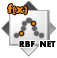

Supplement: Supplementary Data [file bty087_src_code_and_dataset.zip › src_code_and_dataset/java_src/weka/bin/weka/gui/beans/icons/RBFNetwork_animated.gif]

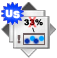

Supplement: Supplementary Data [file bty087_src_code_and_dataset.zip › src_code_and_dataset/java_src/weka/bin/weka/gui/beans/icons/filters.unsupervised.instance.RemovePercentage.gif]

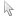

Supplement: Supplementary Data [file bty087_src_code_and_dataset.zip › src_code_and_dataset/java_src/weka/bin/weka/gui/beans/icons/cursor.png]

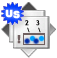

Supplement: Supplementary Data [file bty087_src_code_and_dataset.zip › src_code_and_dataset/java_src/weka/bin/weka/gui/beans/icons/filters.unsupervised.attribute.NumericTransform_animated.gif]

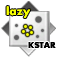

Supplement: Supplementary Data [file bty087_src_code_and_dataset.zip › src_code_and_dataset/java_src/weka/bin/weka/gui/beans/icons/KStar.gif]

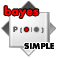

Supplement: Supplementary Data [file bty087_src_code_and_dataset.zip › src_code_and_dataset/java_src/weka/bin/weka/gui/beans/icons/NaiveBayesSimple_animated.gif]

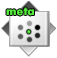

Supplement: Supplementary Data [file bty087_src_code_and_dataset.zip › src_code_and_dataset/java_src/weka/bin/weka/gui/beans/icons/Default_metaClassifier_animated.gif]

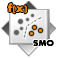

Supplement: Supplementary Data [file bty087_src_code_and_dataset.zip › src_code_and_dataset/java_src/weka/bin/weka/gui/beans/icons/SMO_animated.gif]

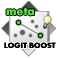

Supplement: Supplementary Data [file bty087_src_code_and_dataset.zip › src_code_and_dataset/java_src/weka/bin/weka/gui/beans/icons/LogitBoost_animated.gif]

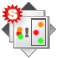

Supplement: Supplementary Data [file bty087_src_code_and_dataset.zip › src_code_and_dataset/java_src/weka/bin/weka/gui/beans/icons/filters.supervised.instance.SpreadSubsample.gif]

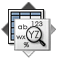

Supplement: Supplementary Data [file bty087_src_code_and_dataset.zip › src_code_and_dataset/java_src/weka/bin/weka/gui/beans/icons/DefaultText.gif]

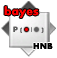

Supplement: Supplementary Data [file bty087_src_code_and_dataset.zip › src_code_and_dataset/java_src/weka/bin/weka/gui/beans/icons/HNB.gif]

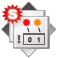

Supplement: Supplementary Data [file bty087_src_code_and_dataset.zip › src_code_and_dataset/java_src/weka/bin/weka/gui/beans/icons/filters.supervised.attribute.NominalToBinary_animated.gif]

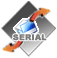

Supplement: Supplementary Data [file bty087_src_code_and_dataset.zip › src_code_and_dataset/java_src/weka/bin/weka/gui/beans/icons/SerializedInstancesLoader.gif]

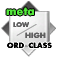

Supplement: Supplementary Data [file bty087_src_code_and_dataset.zip › src_code_and_dataset/java_src/weka/bin/weka/gui/beans/icons/OrdinalClassClassifier_animated.gif]

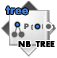

Supplement: Supplementary Data [file bty087_src_code_and_dataset.zip › src_code_and_dataset/java_src/weka/bin/weka/gui/beans/icons/NBTree_animated.gif]

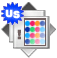

Supplement: Supplementary Data [file bty087_src_code_and_dataset.zip › src_code_and_dataset/java_src/weka/bin/weka/gui/beans/icons/filters.unsupervised.instance.Randomize_animated.gif]

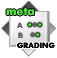

Supplement: Supplementary Data [file bty087_src_code_and_dataset.zip › src_code_and_dataset/java_src/weka/bin/weka/gui/beans/icons/Grading.gif]

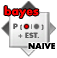

Supplement: Supplementary Data [file bty087_src_code_and_dataset.zip › src_code_and_dataset/java_src/weka/bin/weka/gui/beans/icons/NaiveBayes.gif]

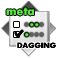

Supplement: Supplementary Data [file bty087_src_code_and_dataset.zip › src_code_and_dataset/java_src/weka/bin/weka/gui/beans/icons/Dagging_animated.gif]

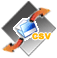

Supplement: Supplementary Data [file bty087_src_code_and_dataset.zip › src_code_and_dataset/java_src/weka/bin/weka/gui/beans/icons/CSVLoader.gif]

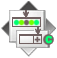

Supplement: Supplementary Data [file bty087_src_code_and_dataset.zip › src_code_and_dataset/java_src/weka/bin/weka/gui/beans/icons/ClassValuePicker.gif]

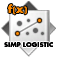

Supplement: Supplementary Data [file bty087_src_code_and_dataset.zip › src_code_and_dataset/java_src/weka/bin/weka/gui/beans/icons/SimpleLogistic.gif]

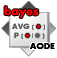

Supplement: Supplementary Data [file bty087_src_code_and_dataset.zip › src_code_and_dataset/java_src/weka/bin/weka/gui/beans/icons/AODE_animated.gif]

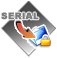

Supplement: Supplementary Data [file bty087_src_code_and_dataset.zip › src_code_and_dataset/java_src/weka/bin/weka/gui/beans/icons/SerializedInstancesSaver_animated.gif]

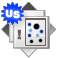

Supplement: Supplementary Data [file bty087_src_code_and_dataset.zip › src_code_and_dataset/java_src/weka/bin/weka/gui/beans/icons/filters.unsupervised.attribute.AddNoise.gif]

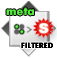

Supplement: Supplementary Data [file bty087_src_code_and_dataset.zip › src_code_and_dataset/java_src/weka/bin/weka/gui/beans/icons/FilteredClassifier_animated.gif]

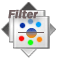

Supplement: Supplementary Data [file bty087_src_code_and_dataset.zip › src_code_and_dataset/java_src/weka/bin/weka/gui/beans/icons/DefaultFilter.gif]

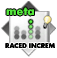

Supplement: Supplementary Data [file bty087_src_code_and_dataset.zip › src_code_and_dataset/java_src/weka/bin/weka/gui/beans/icons/RacedIncrementalLogitBoost.gif]

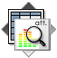

Supplement: Supplementary Data [file bty087_src_code_and_dataset.zip › src_code_and_dataset/java_src/weka/bin/weka/gui/beans/icons/AttributeSummarizer_animated.gif]

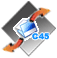

Supplement: Supplementary Data [file bty087_src_code_and_dataset.zip › src_code_and_dataset/java_src/weka/bin/weka/gui/beans/icons/C45Loader_animated.gif]

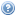

Supplement: Supplementary Data [file bty087_src_code_and_dataset.zip › src_code_and_dataset/java_src/weka/bin/weka/gui/beans/icons/help.png]

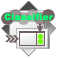

Supplement: Supplementary Data [file bty087_src_code_and_dataset.zip › src_code_and_dataset/java_src/weka/bin/weka/gui/beans/icons/PredictionAppender_animated.gif]
